# Endosphere Microbiome and Metabolic Differences Between the Spots and Green Parts of *Tricyrtis macropoda* Leaves

**DOI:** 10.3389/fmicb.2020.599829

**Published:** 2021-01-11

**Authors:** Yan Wang, Huyin Cheng, Fan Chang, Le Zhao, Bin Wang, Yi Wan, Ming Yue

**Affiliations:** ^1^Microbiology Institute of Shaanxi, Xi’an, China; ^2^College of Pharmacy, Shaanxi University of Chinese Medicine, Xianyang, China; ^3^School of Biological Sciences and Engineering, Shaanxi University of Technology, Hanzhong, China; ^4^College of Chemistry, Biology and Materials Science, East China University of Technology, Nanchang, China; ^5,^ School of Life Sciences, Northwest University, Xi’an, China

**Keywords:** *Tricyrtis macropoda*, microbiome, metabolomics, chlorophyll fluorescence, diversity

## Abstract

**Background:**

Plant leaves are important organs for photosynthesis and biological energy production. The leaves of *Tricyrtis macropoda* have an unusual spotted pattern. However, whether the spots of *T. macropoda* affect the plant microbiome and metabolites is unclear. In this study, we compared differences in the endosphere microbiome and plant metabolites in green parts and spots and the effects of spots on the photosynthesis of leaves.

**Methods:**

16S/ITS sequences and metabolite spectra were obtained by high-throughput amplicon sequencing and ultra-high-performance liquid chromatography–high-resolution mass spectrometry, respectively. Changes in the diversity of the endophytic microbial community and metabolites were studied, and the effect of *T. macropoda* leaf spots on photosynthesis was examined by chlorophyll fluorescence.

**Results:**

The results showed that the relative abundance of *Cercospora* fungi in the leaf spots of *T. macropoda* was significantly higher than that in the green parts (*P* < 0.05) while *Colletotrichum* fungi showed low abundance in the spots. Alkaloid and ketone metabolites were decreased in the green parts compared with the spots, and amino acids, organic acids, lipids, and other compounds were increased in the green parts compared with the spots. A combined analysis of microbial communities and metabolites showed a significant correlation between the endophytic fungal communities and metabolite production. The changes in these metabolites may cause changes in local leaf color. In addition, we found that the spot areas of *T. macropoda* can be photosynthetically normal.

**Conclusion:**

This research showed the relationship between endophytic microorganisms and metabolites, and the findings advance our understanding of endophyte–plant interactions and provide a new direction for investigating the relationship between endophytes and phenotypes.

## Introduction

The leaf spots of *Tricyrtis macropoda* have a peculiar pattern. In the natural environment, the adaxial side of the leaf presents dark brown irregular spots that are usually 5–15 mm in diameter and nearly round, and these leaf spots only occur in three to six leaves of the plant after germination. Leaves far above the ground produce few or no spots (Supplementary Figures 1A,B). In addition, patterns can be formed on the leaves of many angiosperms, such as stripes, spots, or complex designs [e.g., *Orchidaceae* (*Goodyera schlechtendaliana* Rchb. f.), *Liliaceae* (*Drimiopsis kirkii* Baker, *Chlorophytum comosum* f. variegata), *Begoniaceae* (*Begonia cathayana* Hemsl, *Begonia masoniana* Irmsch.), and *Euphorbiaceae* (*Codiaeum variegatum* Juss.)], which are one of the factors that characterize angiosperm biodiversity (Glover, 2014). Moreover, the leaf spots exhibit specific arrangements. Variations in the leaf color in plants will inevitably cause changes in the photosynthetic physiological indices (Wang Z. X. et al., 2016; Chen K. Y. et al., 2018; Du et al., 2019).

*Tricyrtis macropoda* is a perennial herb in the genus *Tricyrtis* Wall in Liliaceae. This plant is found in regions of China, Korea, and Japan in East Asia and mainly distributed throughout forests, grassy areas, or rock crevices in mountainous areas at altitudes of 800–2,400 m (National Pharmacopoeia Commission, 2015). Because of the limited global distribution and lack of a good reference genome for this species, the mechanism of leaf spot formation in *T. macropoda* and its physiological significance in plants are unclear.

Hara (1957) studied the leaf spots of 55 species of plants in 24 families and divided the causes of leaf spots into two categories, each including two types: structural types (including the epidermal type and interstitial type) and pigment types (including the chlorophyll type and pigment type). The structural type of leaf spot results from variation in epidermal cells, causing light interference, diffraction, refraction, and void structures and causing light to reflect twice, with both of these reflections changing the path of incident light on the surface and inside the leaves and then affecting the absorption and reflection spectra of the leaves. These phenomena cause the leaves to appear blue, white, silvery white, light green, or silvery green and form structural leaf spots that affect color (Sheue et al., 2012). Chlorophyll in leaves is an important photosynthetic pigment (Pilar et al., 2016). The chlorophyll type of leaf spot is mainly caused by variations in the chloroplast structure, and the obstruction of chlorophyll synthesis leads to white or yellow leaves. Significant differences are not observed in the tissue structure between leaf spots and the normal green parts of the leaves, although the photosynthetic rate is significantly lower in the leaf spots (Yang, 2015; Li et al., 2017). Finally, the pigment type of leaf spot is caused by anthocyanins, which yield red, purple, and other colors in decorative patterns (Du et al., 2017).

Recent studies have also shown that the leaf color of *Blastus cochinchinensis* Lour. results from a variety of mechanisms, such as epidermal cells, intercellular space, mesophyll cells, chloroplast variation and crystal interaction, which strengthen the white spots in the seedlings (Wang Z. X. et al., 2016; Chen et al., 2017). Gene expression or inhibition often leads to variations in chlorophyll and anthocyanin synthesis (Cho et al., 2016; Gu et al., 2019). Plant microbiota, which is often called the second or extended genome of the host, may directly affect the metabolic activity of plants (Khan et al., 2011; Brader et al., 2014; Huang et al., 2018), and it provides plants with a large number of functional capabilities that can aid in the metabolic processes of host plants encoded by their genomes (Berendsen et al., 2012; Berg et al., 2014; Chen H. H. et al., 2018; Huang et al., 2018). In addition, some microorganisms may infect leaves, thus leading to the formation of plant leaf spots that are often harmful to plants (Khizar et al., 2020; Lin et al., 2020). For example, *Pseudocercospora fuligena* will cause tomato leaves to show melatonin spots (Kang et al., 2019). *Pestalotioid* fungi are one of the major agents underlying leaf spots on mango, and their early foliar symptoms on leaves are small yellow-to-brown lesions. These spots expand with uneven borders, turn from white to gray, and coalesce to form larger gray patches (Shu et al., 2020). *Colletotrichum spaethianum* leads to leaf spots in *Polygonatum odoratum* (Liu et al., 2020). However, the mechanisms of leaf spot formation in *T. macropoda* are currently unclear. Lynch and Hsiao (2019) reviewed the powerful influences of microbial communities associated with animals on host physiology. These microbes regulate metabolism and immune function as well as complex host behaviors. Whether microbial communities associated with plants also affect host physiology, phenotypes, metabolism, and complex immune functions to some extent is of considerable interest.

In this study, the differences in the endophytic microbial community and metabolites between spots and non-spot areas are discussed. We investigated the correlations between the microbiome and metabolites. In this study, we asked three main questions: (1) Are leaf spots related to colonization by microorganisms? (2) Is microbial colonization related to changes in plant metabolites? (3) What is the effect of leaf spots on plant photosynthesis?

## Materials and Methods

### Study Location and Processing of Samples

*Tricyrtis macropoda* was collected from the northern slope of the Qinling Mountains in China at 107°29′40″E, 34°01′38″N and an altitude of 1,644 at 10 m interval. Complete and healthy *T. macropoda* plants were collected and brought back to the laboratory as soon as possible (total of six plant samples). In the laboratory, among the six plant samples, the leaves of each plant sample were divided into three groups (Supplementary Figure 1C). In the first group, the leaves were dark-adapted for 20 min, washed with sterile water to remove surface dust, and placed on a flat tray with the adaxial surface facing upward for a chlorophyll fluorescence experiment. In the second group, the leaves were collected from six samples, washed with sterile water to remove surface dust, separated into spotted and non-spotted parts, and frozen in liquid nitrogen (30 s). After the liquid nitrogen treatment, the tissues were used to extract metabolites. In the third group, the leaves from six samples were collected, washed in 75% alcohol for 2 min, treated with 5% hypochlorite for 3 min, washed with sterile water three times, and cleared of surface microbes. Then, the spotted and non-spotted parts of the leaves were separated and frozen in liquid nitrogen (30 s). After the liquid nitrogen treatment, total DNA was extracted with a DNA extraction kit. All samples and backup samples were stored at –80°C for further experiments.

### DNA Extraction and Sequencing

DNA was extracted from 100-mg samples from the spotted and non-spotted parts of the *T. macropoda* leaves using magnetic beads and a plant genomic DNA extraction kit (Tiangen Plant Genomic DNA Extraction Kit DP342) following the kit instructions. The internal transcribed spacer regions of the fungal ribosomal RNA gene were amplified by PCR using the primers ITS1-1F-F CTTGGTCATTTAGAGGAAGTAA and ITS1-1F-R GCTGCGTTCTTCATCGATGC (Xiong et al., 2016). The bacterial 16S ribosomal RNA genes were amplified by PCR using the primers 341F-CCTAYGGGRBGCASCAG and 806R-GGACTACNNGGGTATCTAAT (Charlotte et al., 2014). PCR was carried out using a 20-μl mixture containing 4 μl of 5 × FastPfu buffer, 0.8 μl of primer (5 mm), 2 μl of 2.5 mM dNTPs, 0.4 μl of Fast Pfu polymerase, and 10 ng of template DNA. The amplification products were extracted from 2% agarose gel, and the AxyPrep DNA gel extraction kit (Axygen Bioscience, United City, CA, United States) was used. Purifications were carried out according to the manufacturer’s instructions and quantified by QuantiFluor-St (Promega, Durham, NC, United States).

The purified PCR products were measured by Qubit 3.0 (Life Invitrogen, Waltham, MA, United States). The Illumina library was constructed using polymerized DNA products according to the preparation process of the Illumina genomic DNA library. The amplified library was paired and sequenced on the Illumina MiSeq platform (Beijing Novosource Bioinformation Technology Co., Ltd., Beijing, China) according to the standard protocol. The original data are stored in the National Center for Biotechnology Information (NCBI) sequence and the archived database (SRA: SAMN14490841), and they are accessible via the link https://www.ncbi.nlm.nih.gov/biosample/14490841.

### Sequence Processing

Using the analytical platform of the research center, the original 16S sequences were first obtained by FastQC software, the sequences of fungi less than 200 bp and bacteria less than 400 bp in length were filtered, and the primers were deleted by the Cutadapt 1.18 program. Then, Usearch (version 11)^1^ was used for follow-up analysis of biological information. The fastq_mergepairs command of Usearch was used to merge paired end sequences, the fastq_filter command was used to control sequence quality, the Unoise3 algorithm was used for operational taxonomic unit (OTU)-like (sub-OTU) non-parametric clustering, and the fastx_uniques command was used to remove redundant and singleton sequences (the minimum parameter was eight). After removing chimeras with Usearch (Edgar, 2010), the similarity of OTUs was 97%. An OTU table was generated. After clustering, the sequences were annotated with the UNITE database, and a cutoff value of 0.8 obtained by the Sintax method was used. The OTU table was constrained by using the smallest number of sequences in the grouping. Annotation was performed with the Ribosomal Database Project (RDP) and UNITE reference databases (v7.1) for the bacterial and fungal communities, respectively, (Abarenkov et al., 2010). Mitochondrial, chloroplast, plant, and protoplast entries were deleted, and the unclassified contaminant sequences were filtered out. There were 916 OTU sequences in the final community data set. After analyzing the complete data set, the leaf spots and green parts were separated to assess the differences between them.

### Microbiome Statistical Analysis

Statistical analyses were performed using R 3.5.1 (R Foundation for Statistical Computing, Vienna, Austria) (30). The parameters of alpha diversity and beta diversity were calculated by Usearch (Lozupone et al., 2006). Analysis of variance (ANOVA) was performed to analyze the overall differences, Student’s *t*-test was used to analyze the differences between groups, and the differences in the alpha diversity index between leaf spots and the green part of leaves were studied. Beta diversity was calculated using the binary Jaccard algorithm for principal coordinate analysis (PCoA) and then visualized using PERMANOVA ordinations to illustrate compositional differences. The unweighted pair group method with arithmetic mean (UPGMA) procedure was used in the cluster analysis to measure the evolutionary distances between samples. Venn diagrams were used to show the numbers of common and unique OTUs among samples (Hanbo and Paul, 2011) and intuitively visualize the coincidence of OTUs among samples. The error rate of each type of ANOVA model was corrected by the false discovery rate (FDR). Quantitative Insights Into Microbial Ecology (QIIME) software was used to select the OTU sequence with the highest abundance at the taxonomic level for the species analysis to determine the frequency of bacteria and fungi in different parts of the leaves.

### Metabolite Extraction

Fifty milligrams of each sample were weighed, and then 1,000 μl of extract [methanol:acetonitrile:water = 2:2:1 (V/V)] was added. The samples were vortexed for 30 s, homogenized at 40 Hz for 4 min, and sonicated for 5 min in an ice-water bath. The homogenization and sonication cycle was repeated three times, followed by incubation at −20°C for 1 h and centrifugation at 12,000 rpm and 4°C for 15 min. The resulting supernatants were transferred to liquid chromatography–mass spectrometry (LC–MS) vials and stored at −80°C until ultra-high-performance liquid chromatography (UHPLC)–quadrupole/electrostatic field (QE) Orbitrap/MS analysis was performed. The quality control (QC) sample was prepared by mixing equal aliquots of the supernatants from all of the samples (Doppler et al., 2016) and used for the metabolomic analysis.

### Metabolite Profiling

LC-MS/MS analyses were performed using a UHPLC system (1,290, Agilent Technologies) with a UPLC HSS T3 column (2.1 mm × 100 mm, 1.8 μm) coupled to a Q Exactive instrument (Orbitrap MS, Thermo). Mobile phase A was 0.1% formic acid in water (positive mode) and 5 mmol/L ammonium acetate in water (negative mode), and mobile phase B was acetonitrile. The elution gradient was as follows: 0 min, 1% B, 1 min, 1% B, 8 min, 99% B, 10 min, 99% B, 10.1 min, 1% B, and 12 min, 1% B. The flow rate was 0.5 ml/min (16 min, 1% B), and the injection volume was 3 μl. A QE mass spectrometer was used to acquire MS/MS spectra on an information-dependent basis (IDB) during the LC/MS experiment. In this mode, the acquisition software (Xcalibur 4.0.27, Thermo) continuously evaluated the full-scan survey MS data during data collection and triggered the acquisition of MS/MS spectra. Electrospray ionization (ESI) source conditions were set as follows: sheath gas flow rate of 45 Arb, aux gas flow rate of 15 Arb, capillary temperature of 400°C, full MS resolution of 70,000, MS/MS resolution of 17,500, collision energy of 20/40/60 eV in the chemical non-equilibrium (NCE) model, and a spray voltage of 4.0 kV (positive mode) or −3.6 kV (negative mode) (Wang J. L. et al., 2016).

### Data Preprocessing and Annotation

To explore the composition of the metabolites of the leaf spot areas of *T. macropoda*, Simca software (v15.0.2, Sartorius Stedim Data Analytics AB, Umeå, Sweden) was used to process the data via log conversion and centralized treatment, and automatic modeling was then carried out to perform principal component analysis (PCA) (Wiklund et al., 2008). The raw data were converted to mzXML format using Proteo Wizard. MAPS software (version 1.0) was used to correct the retention time, mass-to-charge ratio (*m*/*z*), peak intensity, peak extraction, peak integral, and peak alignment. An in-house MS2 database and R were used for metabolite identification.

### Multivariate Statistical Analysis

The peak mass intensity of each sample was normalized and Pareto-scaled with Simca P software (version 12.0, Umetrics, Umeå, Sweden). PCA and orthogonal partial least squares discriminant analysis (OPLS–DA) were used to study the differences in metabolite composition among 12 samples (2 leaf parts × 6 biological replicates). The first component was used to extract the reliability [*P*(corr)] value of all metabolites in the OPLS–DA. We selected metabolites satisfying the following criteria as potential markers: (1) high confidence [| *P*(corr)| > 0.6] in discrimination between the spots and green parts of leaves, (2) mean intensities in leaf spots that were significantly different from those in the green parts of leaves (*P* < 0.05), and (3) a minimum two-fold change in level between leaves and green spots. The *P*-value was calculated using an independent two-sample *t*-test.

### Integrative Analysis of the Metabolome and Microbiome

Based on the endophyte community annotation, at the genus level, the fungal community members with an abundance greater than 0.5% and the identified differential metabolites were screened. A correlation analysis was carried out using the Spearman algorithm, and the correlation *P*-value was less than 0.05. Based on these results, the relationship between the microbial and metabolite groups was determined by visualization in R.

## Results

### Metabolic Differences Between the Spots and Green Parts of Leaves

**FIGURE 1:**
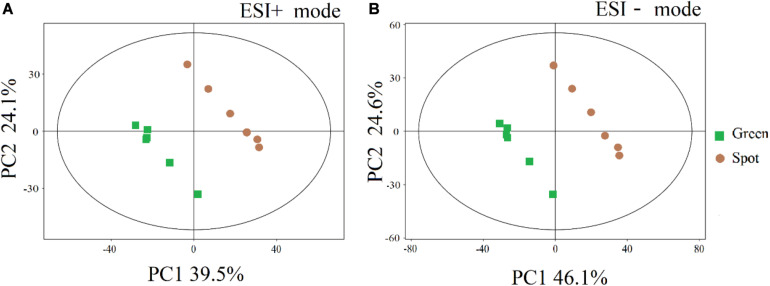
PCA scores of different parts of the leaves. **(A)** Electrospray ionization positive mode. **(B)** Electrospray ionization negative mode. The abscissa PC1 and ordinate PC2 represent the scores of the first and second principal components, respectively, and the color and shape of points represent the experimental groups of samples. All samples are within the 95% confidence intervals.

To compare the metabolite pattern between the spots and green parts, we first performed a PCA for the features obtained in positive ion mode. The cumulative amount of variation explained by the *X* variable (PC1) was 39.5%, while that explained by the *Y* variable (PC2) was 24.1% (Figure 1), and the corresponding values for the results obtained in negative ion mode were 46.1 and 24.6%, respectively. Furthermore, the spots and green parts of leaves were significantly separated by the PCA (Figure 1), which showed that the composition of metabolites was different. In addition, a plot of OPLS-DA scores was used to examine the difference between the two parts (Supplementary Figures 2A,B), and the prediction value of the model was more than 95%. The OPLS-DA permutation test similarly proved this separation (Supplementary Figure 2C,D).

### Screening of Differential Metabolites

**FIGURE 2:**
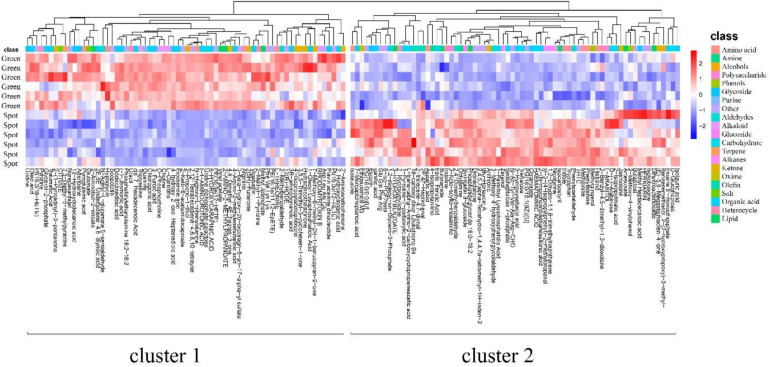
Metabolite patterns detected in two clusters. The abscissa represents the different experimental groups, the ordinate represents metabolites compared between the groups, and the colored blocks at different positions represent the relative expression of metabolites at those positions. The metabolites for which VIP > 1 and *P* < 0.05 were considered to be significantly changed.

Based on the inherent characteristics of QE metabolomics data, 28,570 mass features were measured and 527 (positive 351, negative 176) metabolites were identified from mass spectrometry. We screened 450 different metabolites with significant differences (*P* < 0.05). There were 244 upregulated species and 196 downregulated species (Supplementary Figure 3). The detected differential metabolites often had similar/complementary results and biological functions or were positively/negatively regulated by the same metabolic pathway, thus leading to similar or opposite expression characteristics between experimental groups. We identified 138 metabolites with such characteristics (Supplementary Table 1). A hierarchical cluster analysis of identified substances and a quantitative calculation with a Euclidean distance matrix were carried out. The metabolites with the same characteristics were classified into one group. Hierarchical clustering of the metabolite patterns revealed two clusters (Figure 2). In the first cluster, most of the alkaloids (5/7) and ketones (8/11) were detected. These ions were less abundant in the spots than in the green parts. In addition, 5 polar amino acids, 20 organic acids, and 4 lipids were less abundant in the spots than in the green parts (Supplementary Table 2). The second cluster was mainly composed of amino acids (6 types), aldehydes (4 types), sugars (4 types), organic acids (16 types), and lipids (5 types). These ions were more abundant in the spots than in the green parts. The dynamic changes in these metabolites may be related to changes in metabolic pathways, and the significantly increased metabolites may be the cause of the change in leaf color.

### Analysis of Microbial Community Diversity in Spotted and Non-Spotted Parts

**FIGURE 3:**
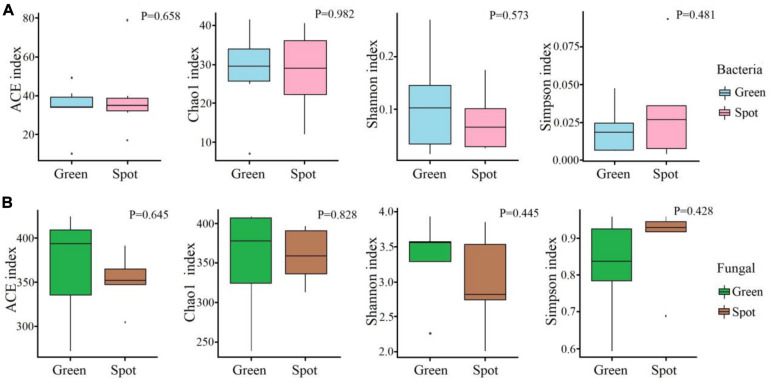
Alpha diversity of the microbiome communities in different parts. **(A)** Alpha diversity index of the bacterial communities. **(B)** Alpha diversity index of the fungal communities. The *t*-test method was used to study the alpha diversity. The box diagram shows the first (25%) and third (75%) quartiles, median values, and maximum and minimum observational values in each data set. The alpha diversity estimation is presented for the samples of green areas and spots on leaves.

We analyzed the diversity and community composition of bacteria and fungi in different parts of the leaves (spotted and non-spotted parts). We found similar bacterial species richness (*P* = 0.658) and fungal species richness (*P* = 0.645) between the spotted and non-spotted parts. Similarly, alpha diversity parameters (ACE, Chao1, Shannon, and Simpson indices) of the microbiome of the green leaf parts and spots were not significantly different. The abundances were also similar between the two parts of leaves. We also calculated the community diversity of the two groups of samples. The fungal and bacterial diversities in the green parts were greater than those in the spots (Figure 3).

**FIGURE 4:**
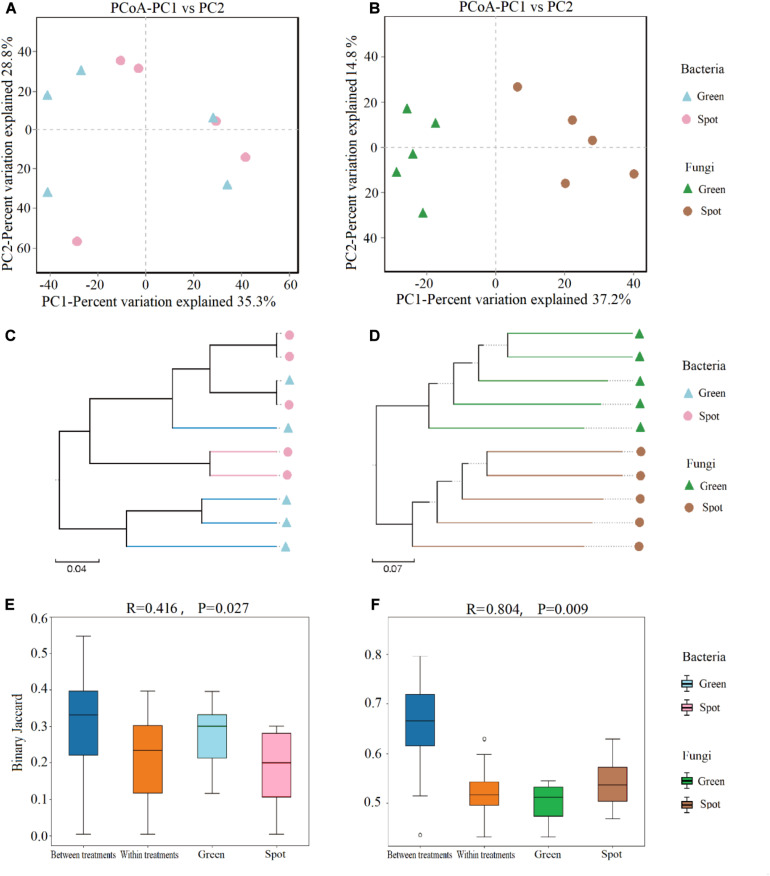
Analysis of endophytic microbial community diversity in leaves. **(A)** PCoA of the bacterial community at the OTU level based on the binary Jaccard algorithm. The horizontal and vertical coordinates are the two characteristic values that explain most of the variation between samples, and the amount of variation explained is expressed as a percentage. **(B)** PCoA of the fungal community at the OTU level based on the binary Jaccard algorithm. Each OTU is shown as a vector in the PCoA map, and the fungal communities of the green parts and spots are distinct. **(C)** UPGMA hierarchical clustering of bacteria. Based on the binary Jaccard algorithm, UPGMA hierarchical clustering of different samples was carried out. Closer samples correspond to shorter branch lengths, which indicates that the species composition of the two samples is more similar. **(D)** UPGMA hierarchical clustering of fungi. **(E)** Beta distance data for bacteria, based on the binary Jaccard algorithm. **(F)** Beta distance data for fungi.

We also evaluated the beta diversity of endophytes in the spotted and non-spotted parts of the leaves, compared and determined the composition of the endophytes in the different parts of leaves, calculated a binary Jaccard dissimilarity matrix, and showed the overall similarity in microbial community structure among the samples by PCoA (Figures 4A,B). In addition, we used the UPGMA cluster analysis to reveal changes in community composition (Figures 4C,D). The PCoA showed no significant clustering of the bacterial community between the spotted and non-spotted parts (Figure 4A), although the fungal community displayed stronger clustering (Figure 4B). At the OTU level, PC1 explained 37.2% of the total variation, PC2 explained 14.8%, and the cumulative variance explained by the two variables was 52%. Hierarchical clustering of the samples was based on the binary Jaccard dissimilarity values, which were superimposed on the PCoA plot. The hierarchical clustering of fungi (at the OTU level) revealed complete clustering. To support the clustering results of the leaf fungal community obtained via the PCoA, an analysis of similarities (ANOSIM) was performed, and it indicated a significant difference between leaf spots and non-spots areas (*R* = 0.804, *P* = 0.009) (Figures 4E,F).

### Differences in Microbial Community Composition Between the Spotted and Non-Spotted Parts

**FIGURE 5:**
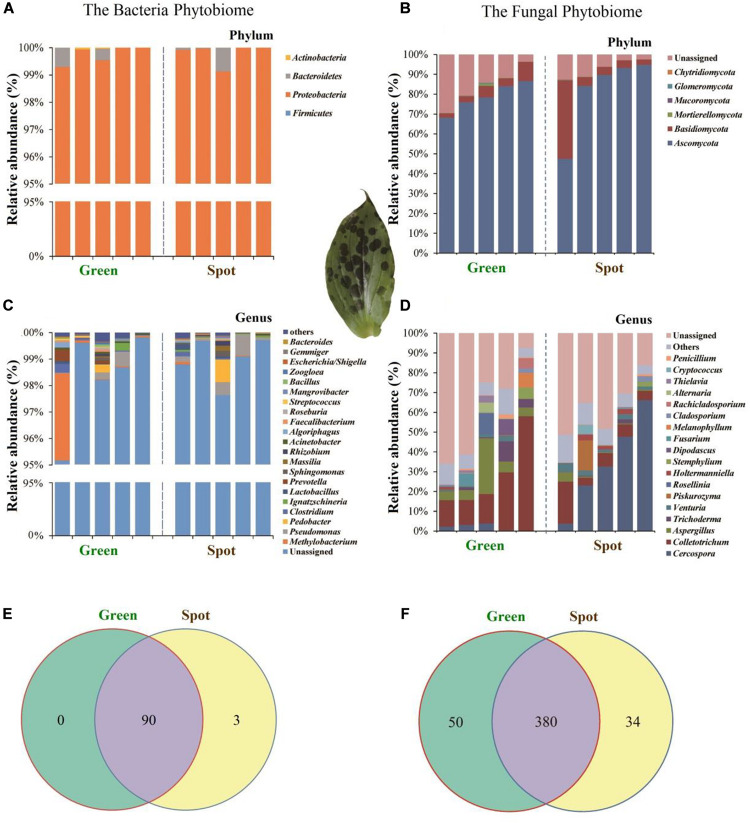
Distribution of species at different classification levels. Selected species with an abundance greater than 0.5% are displayed in the figure and combined other species into “Others”; “Unassigned” represents the species without taxonomic annotation. Different microbial communities are distinguished by different color combinations. The Venn diagram shows the numbers of OTUs in two leaf parts, with the number shown in the overlapping part of the diagrams indicating the total number of OTUs shared between the two leaf parts and the numbers shown in the non-overlapping parts indicating the numbers of unique OTUs in each part. **(A)** Relative abundance of bacteria at the phylum level. **(B)** Relative abundance of fungi at the phylum level. **(C)** Relative abundance of bacteria at the genus level. **(D)** Relative abundance fungi at the genus level. **(E)** Venn diagram of bacteria. **(F)** Venn diagram of fungi.

In this study, we analyzed the division of fungi and bacteria at different levels. The results revealed that the bacteria in the green and spotted areas were mainly Proteobacteria (green 99.49%, spot 99.63%) (Figure 5A) while the fungi were mainly concentrated in Ascomycota (80.27%), Basidiomycota (7.92%), and Mortierellomycota (0.22%) (Figure 5B). There was no significant difference in the species of dominant organisms (fungi and bacteria) between the spotted and non-spotted parts. In addition, we analyzed the differences in microorganisms at different levels. Bacteria did not exhibit significant differences between the two parts (Figures 5A,C). However, for fungi at the genus level (Figure 5D), *Cercospora* exhibited a higher relative abundance (34.66%) in the leaf spots than in the green parts (*P* = 0.015). In addition, at the genus level, *Colletotrichum* fungi were less abundant in the spots (25.68% in the spots and 8.62% in the non-spot area), which indicated that symbiosis with *Colletotrichum* fungi in the spot areas may have been inhibited. To better show the distribution of microbial differences in plant leaves, we calculated the proportion of OTUs in specific areas of plant leaves and the OTUs shared by different areas (Figures 5E,F). For fungi, a total of 7.33% of the OTUs were unique to the spots and 10.78% were unique to the green parts. However, bacteria had few such differences (Figure 5E).

### Combined Analysis of Microbial and Metabolite Groups

**FIGURE 6:**
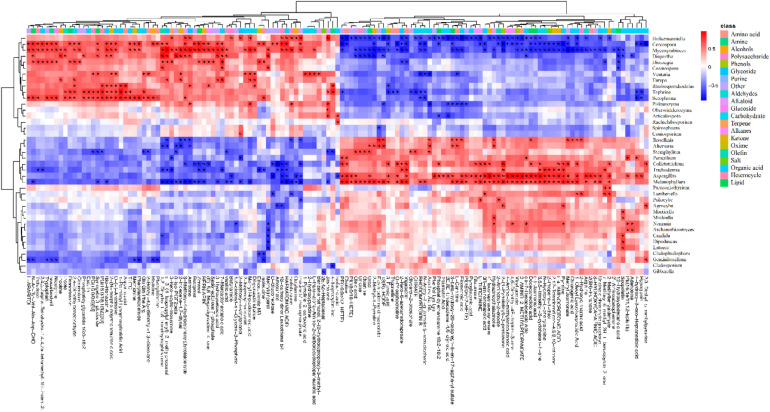
Heat map of the correlations between plant metabolites and the endophyte community. The metabolites were significantly related to 40 genera of endophytic fungi belonging to 3 phyla and 11 classes. Red indicates a positive correlation between these metabolites and the microbes, blue indicates a negative correlation, and white indicates a non-significant correlation (correlation = 0). The data with a correlation *P*-value less than 0.05 are marked with “#.” The abscissa shows the metabolites, and the ordinate shows the taxa.

At the genus level, we used the Spearman algorithm to calculate correlations between fungal community members with an abundance greater than 0.5% and the identified differential metabolites (Supplementary Figure 5). Then, we calculated *P-*values for the correlations. The data with a correlation *P*-value less than 0.05 are shown in Figure 6, which reveals that the endophytic fungi significantly related to the differences in metabolites (correlation *P* < 0.05) were mainly *Cercospora* and *Diaporthe* of Ascomycota, *Holtermanniella* and *Dioszegia* of Basidiomycota, and related taxa (see Supplementary Table 2 for additional classification information). In the previous analysis, the abundance of endophytic fungi in leaf spots was also different from that in green parts. Thus, there is a close relationship between plant metabolites and endophytes, which may be caused by the production of metabolites by the endophytes, an influence on host secondary metabolite production, or more complex host–microorganism interactions.

### Analysis of Chlorophyll Fluorescence Parameter Differences

**FIGURE 7:**
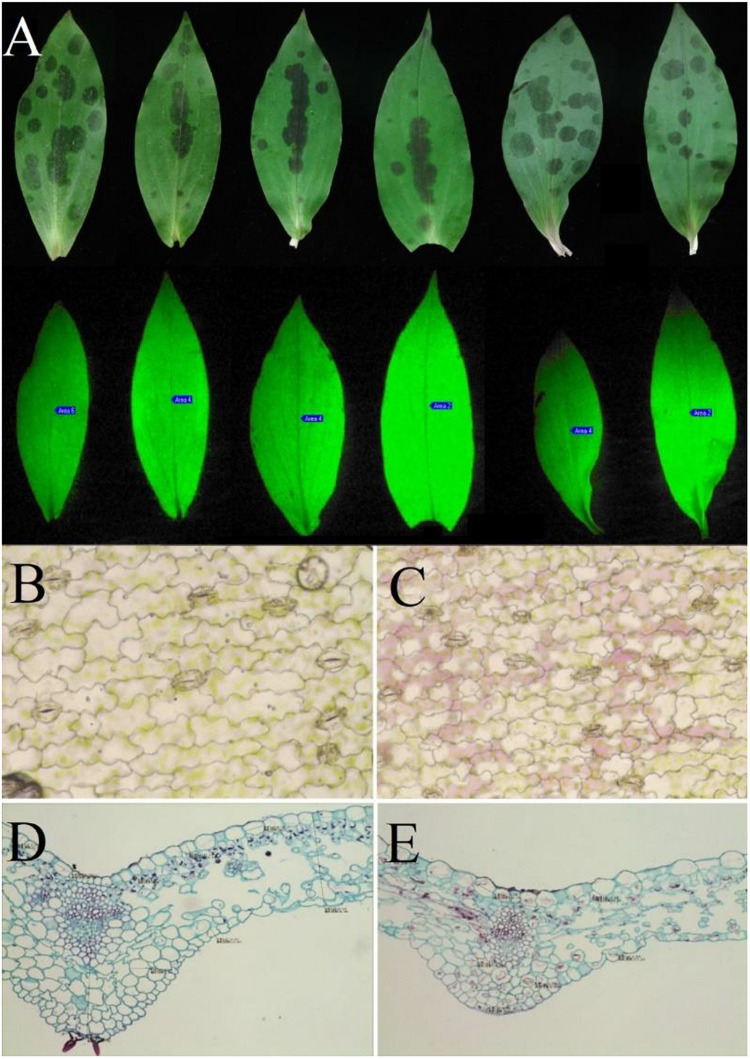
Optical images of the leaves of *T. macropoda*. **(A)** Image of fluorescence on the blade of *T. macropoda*. **(B)** Microscopic image of a cross-section of the green part of the blade, with a magnification of 16 × 10. **(C)** Microscopic image of a cross-section of a spot on the blade, with a magnification of 16 × 10. **(D)** Microscopic image of a longitudinal section of the green part of the blade, with a magnification of 10 × 10. **(E)** Microscopic image of a longitudinal section of a spot on the blade, with a magnification of 10 × 10.

F0 is the fluorescence yield when photosystem II (PSII) reaction centers are completely open in the dark-adapted state. The value of F0 is closely related to the light-catching antenna system, the state of PSII reaction centers (Sun et al., 2015), and the concentration of chlorophyll (Shasmita et al., 2019; Zhang, 2019). After dark adaptation, the initial fluorescence (F0), maximum fluorescence (Fm), and variable fluorescence (Fv) were obtained at the onset of illumination. The results showed significant differences in F0 and Fm between the leaf spots and non-spot parts (*P* < 0.001). The F0 value of leaf spots and non-spot parts decreased by 19.6%, and the Fm value decreased by 17.91%. Significant differences were not observed in PSII potential activity (Fv/F0) or the maximum quantum efficiency of PSII photochemistry (Fv/Fm) between the spotted and non-spotted parts of leaves (*P* = 0.77 and *P* = 0.532, respectively) (Table 1). Moreover, significant differences were not observed in the fluorescence decay index (FDI) between the spotted and non-spotted parts (*P* = 0.36), which indicated that the formation of leaf spots had no significant effect on the photosynthetic capacity of the leaves (Figure 7A). In addition, micrography revealed the structural integrity of the spotted tissue (Figures 7B–E).

**TABLE 1:** Different chlorophyll fluorescence parameters in different leaf regions of *T. macropoda*.

	**Green**	**Spot**	***P*-value**
F0	599.09247.02	481.41203.85	0.001**
Fm	2833.177987.19	2325.74934.60	0.001**
Fv	2234.08740.16	1844.34775.54	0.001**
Fv/Fm	0.790.05	0.790.05	0.777
Fv/F0	3.760.71	3.821.05	0.532
FDI	1.160.83	1.270.89	0.370

Spotted and non-spotted parts of *T. macropoda* leaves subjected to chlorophyll fluorescence experiments. After dark adaptation, the initial fluorescence parameter (F0), maximum fluorescence parameter (Fm), variable fluorescence parameter (Fv), fluorescence decay index (FDI), PSII potential activity (Fv/F0), and maximum photosynthetic efficiency (Fv/Fm) of the two regions were obtained and the *t-*test method for significant differences was performed between the two regions. Significance levels: **P ≤ 0.001. The results are presented graphically in Supplementary Figure 2.

## Discussion

The formation of pigment patterns depends on pigment (metabolite) biosynthesis in cells (Davies et al., 2012). In this study, we examined the different metabolites between the spot and green parts of *T. macropoda* leaves. We screened 527 types of metabolites whose VIP value was more than 1 in the OPLS-DA model results as well as 68 kinds of metabolites that were decreased in the spots of leaves. In addition, 70 metabolites were significantly upregulated, including 6 amino acids (including tryptophan), 4 aldehydes, 4 sugars, 16 organic acids, and 5 lipids. Regarding the analysis of plant metabolites, the metabolites of the same cluster were also significantly different between spots and non-spots, and the change in these metabolites may be the cause of the change in leaf color.

To investigate whether the endosphere microbiome is uniformly colonized in leaf spots and non-spots areas, we studied the endosphere microbiome in spotted and non-spotted areas by high-throughput sequencing. As shown in the box plot (Figure 3), the results indicated that the α diversity of the endosphere microbiome in the spotted and non-spotted areas was not significantly different. Also, no significant differences in the beta diversity of the bacterial endophytes were found between the two leaf parts. As regards the β diversity of endophytic microorganisms in leaves, there was no significant difference in the β diversity of endophytic bacteria, but in our study, Proteobacteria were significantly enriched in the leaf. This result is different from previous studies. Proteobacteria have different enrichment abundances in different plants, which may be due to the effect of plant genotypes on bacterial colonization (Cregger et al., 2018) or may be caused by primer bias. Further study is required to verify this result. In contrast, the β diversity of endophytic fungi was significantly different, which may be caused by the uneven colonization of endophytic fungi in leaves. In the annotation of microbial species, we found differences in species diversity and abundance between the leaf spots and green parts. At the phylum level, Ascomycota was the main fungal colonizer of leaves, and this phylum is considered to be the most common group of endophytes in plants (Guo, 2016). Ascomycota is also widely found in other plants, such as grasses, flowers, and crops (Ingrid et al., 2007; Joseph et al., 2015; Stephane et al., 2015). At the genus level, *Cercospora*, *Colletotrichum*, and *Aspergillus* in Ascomycota accounted for 40.88% of the endophytic fungi in this study (Figure 5D) and differed significantly in abundance between the two leaf parts (*P* < 0.05). *Cercospora* was significantly more abundant in the spots than in the green parts (*P* < 0.05). *Cercospora* is believed to cause the formation of leaf spots in plants and eventually lead to leaf spot disease (Albert and Charles, 1950; Heng et al., 2020). *Cercospora* can cause necrotic damage to the leaves, thus leading to suborbicular, oil-stained brown spots on the leaf surface (Chupp, 1954; Xie et al., 2017), and *Cercospora* can cause frog eye spots in cigar tobacco in Hainan (Zhao et al., 2020). Therefore, we speculate that the formation of leaf spots may be closely related to the colonization of leaves by fungi.

Because microorganisms can regulate plant immunity and affect plant metabolism (Lee and Mazmanian, 2010; Lebeis et al., 2015; Beckers et al., 2017), the relationship between microbes and metabolites was studied. We used a thermograph to show the relationships between microorganisms and metabolites in different leaf regions and calculated the *P*-values of the correlations between endophytic fungi and differential metabolites. The results showed significant correlations of *Cercospora* and *Diaporthe* in Ascomycota and *Holtermanniella* and *Dioszegia* in Basidiomycota with 118 different metabolites. This result was consistent with the different microbes observed between the two parts; therefore, it can be preliminarily inferred that colonization by endophytic fungi may play a role in changes in plant metabolites. To date, Arabidopsis, rice, corn, and other model plants have been studied in detail. A study on the Arabidopsis endophytic microbiome described the root and leaf microbial communities and explored the function of the host microbiome. Microbiota specializations have their own functional capabilities to their respective niche (Bai et al., 2015). A study on the functional characteristics of the endophytic community of rice roots showed that plant endophytes may participate in the metabolic processes of rice (Vain et al., 2014). Recent research has shown that *Salvia miltiorrhiza* has a unique microbial community that is rich in functions related to secondary metabolism. These microorganisms can aid in the metabolic processes encoded by the host plant genome. The interactions between *S. miltiorrhiza* and endophytes can enhance the biomass production of the plant and may also affect the tanshinone pathway (Chen H. H. et al., 2018; Huang et al., 2018). This result suggests that different microbial communities can cause differences in the metabolites in *S. miltiorrhiza*. It can be preliminarily speculated that the uneven colonization of endophytic fungi may have an effect on the changes in plant metabolites and the relationships between metabolites and phenotype may be mediated by changes in the composition of the microbiome. The impact of microorganisms on the metabolic pathways, functions, and dynamics of host plants requires further study.

In addition, to determine whether differential colonization by fungi affects the photosynthesis of plants or causes damage to leaves, we measured the chlorophyll fluorescence parameters of leaves, which can not only characterize photosynthesis but also reflect the intrinsic characteristics of photosynthesis (Wang et al., 2019). For the determination of chlorophyll fluorescence parameters of *T. macropoda* leaves, the values of F0 and Fm were obtained. F0 is the parameter describing the dark adaptation of leaves when their reaction centers are fully open, although it does not characterize the state of photochemical reactions. F0 is related to chlorophyll concentration and indicates the activity of the photosystem II (PSII) center (Lu et al., 1994), and a decrease in the F0 value indicates an increase in the heat dissipation of antenna chlorophyll in the leaf (Nan and Lin, 2019; Zhou et al., 2019). An F0 increase indicates that the PSII reaction center was damaged (Xu et al., 1999). In this study, a decrease in the F0 values indicates an increase in plant heat dissipation or a decrease in chlorophyll concentration, both of which may reduce light absorption in plant leaves. Bauer found that the photosynthetic rate of seedling leaves of ivy was lower than that of mature leaves and that the adaptability of seedlings to strong light was weaker than that of mature leaves (Bauer and Bauer, 1980). *T. macropoda* leaf spots occur in only three to six leaves of the plant after germination, and leaves far above the ground produce a small amount of spots, if any. *T. macropoda* germinate in early spring when trees and other tall shrubs have not yet formed shaded environments. During this period, the leaf spots may increase as a result of plant heat dissipation or a decrease in chlorophyll concentration, which may enable self-protection against damage by strong light (Brugnoli et al., 1998; Wiklund et al., 2008). This protection mechanism will be investigated in the future. Fv/Fm is the maximum quantum efficiency of PSII photochemistry, and it represents the activity of the PSII centers (Wungrampha et al., 2019). The values of Fv/Fm and Fv/Fo vary very little under non-stress conditions (Shirke and Pathre, 2003; Kumud et al., 2011), and the Fv/Fm or Fv/F0 values between spots and non-spots areas were not significantly different (Table 1), which also indicated that uneven colonization of leaves by fungi did not cause stress effects on the plants. In addition, the chloroplast structure of the spots was complete. We speculate that the leaf color pattern may enable *T. macropoda* to adapt to the light environment in the initial stage of growth. Additionally, while ensuring normal photosynthesis, the spots may reduce leaf damage caused by strong light by increasing heat dissipation. Hence, the leaf color pattern may help *T. macropoda* to be well adapted to strong-light environments in the seedling stage, which is likely the result of long-term coevolution between plants and microorganisms.

## Conclusion

A comprehensive study of the microbiomics and metabonomics of the spots and green parts of leaves was performed. The results showed that the bacterial diversity of green leaf parts and spots was not significantly different and that the diversity of endophytic fungi and metabolites was different in spotted and non-spotted areas. The enrichment or depletion of 118 metabolites was correlated with the occurrence and abundance of four fungi in the two leaf parts, and the results showed that some microorganisms were significantly related to certain types of metabolites. The results showed significant correlations of *Cercospora* and *Diaporthe* in Ascomycota and *Holtermanniella* and *Dioszegia* in Basidiomycota with 118 different metabolites. In addition, studies of chlorophyll fluorescence have shown that these leaf spots conduct normal photosynthesis; thus, this leaf color pattern may enable *T. macropoda* to be well adapted to strong-light environments in the seedling stage. Our research provides new insights into the relationship between endophytic microbes and plant phenotypes and emphasizes the effectiveness of comprehensive methods used to understand this process.

## Data Availability Statement

The datasets presented in this study can be found in online repositories. The names of the repository/repositories and accession number(s) can be found in the article/Supplementary Material.

## Author Contributions

YaW and MY: conceptualization, writing—review and editing, and funding acquisition. MY: methodology. FC and LZ: software, formal analysis, and visualization. BW, LZ, and YiW: validation. YaW and HC: investigation. FC: the data curation. YaW: writing—original draft preparation. WY: project administration. All authors have read and agreed to the published version of the manuscript.

## Conflict of Interest

The authors declare that the research was conducted in the absence of any commercial or financial relationships that could be construed as a potential conflict of interest.

## Footnotes

1 http://www.drive5.com/usearch/
