# Supplementary figures and images for: Endosphere Microbiome and Metabolic Differences Between the Spots and Green Parts of Tricyrtis macropoda Leaves
Source: Front Microbiol. 2021 Jan 11;11:599829. doi: 10.3389/fmicb.2020.599829 (PMC7829350; doi:10.3389/fmicb.2020.599829)

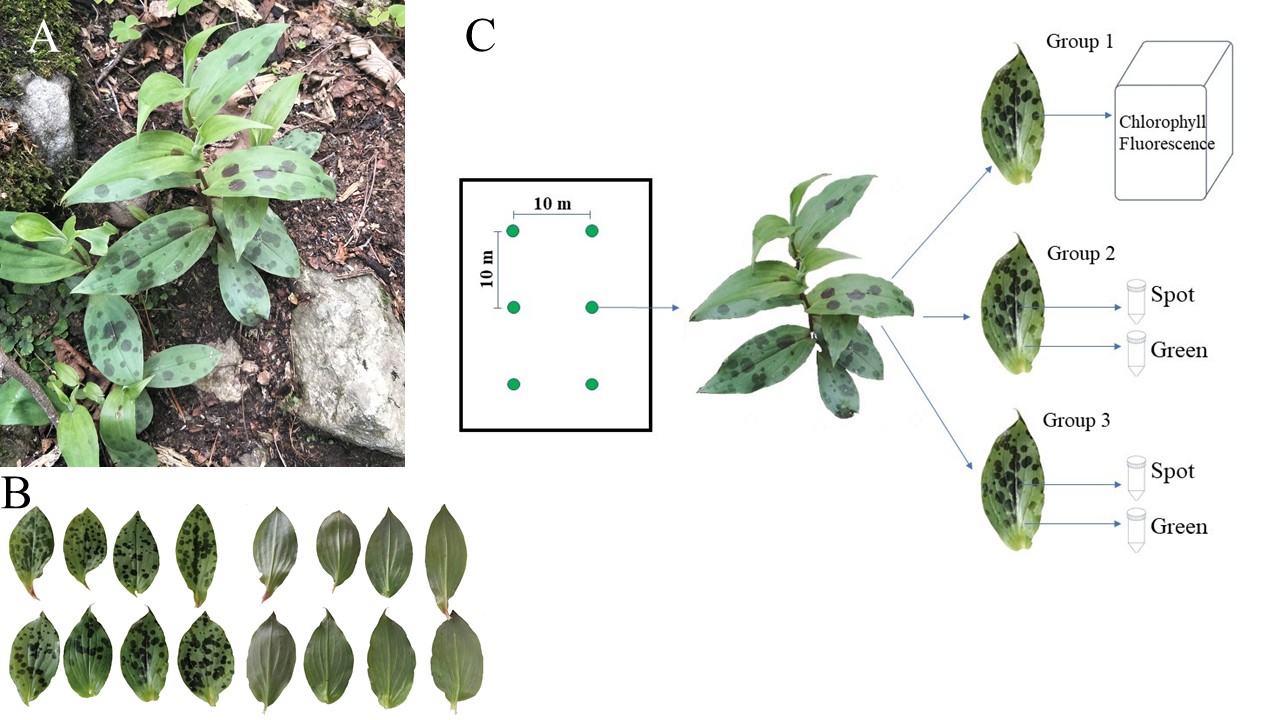

Supplement: Supplementary file 1 [file Image_1.JPEG]

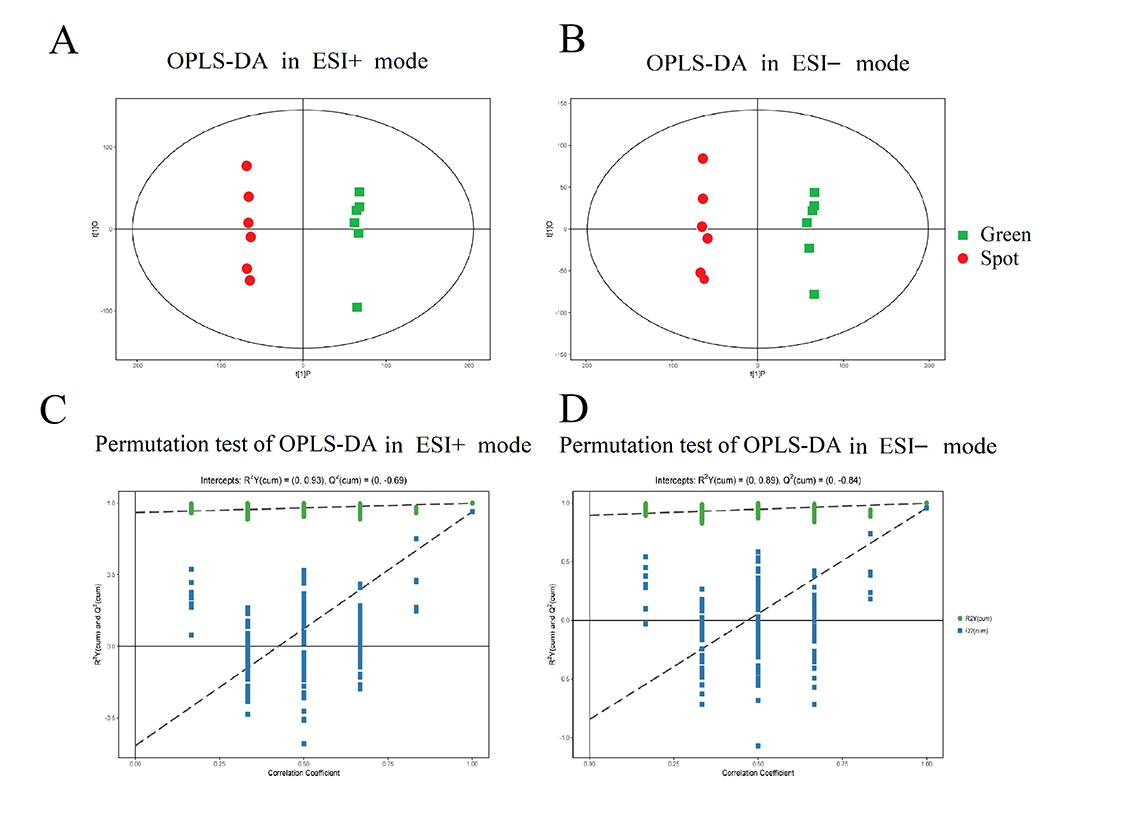

Supplement: Supplementary file 2 [file Image_2.JPEG]

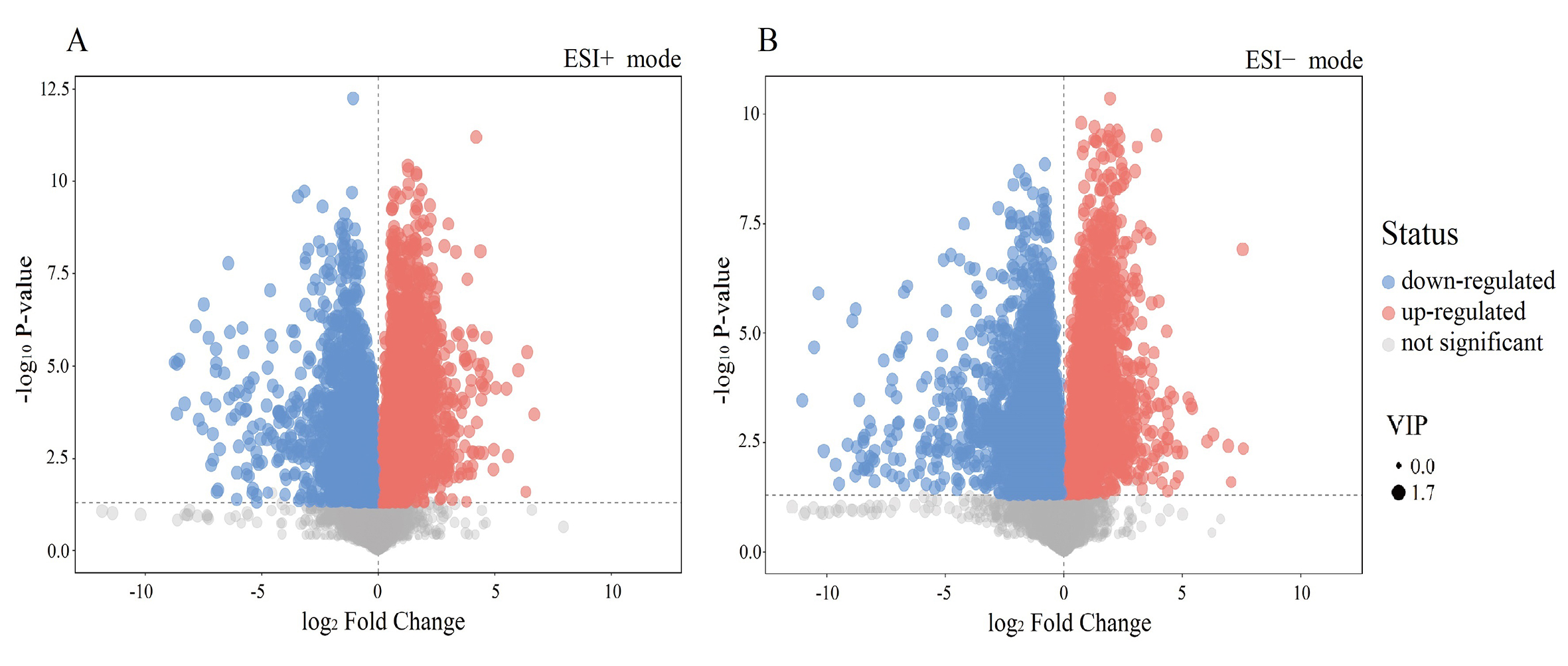

Supplement: Supplementary file 3 [file Image_3.JPEG]

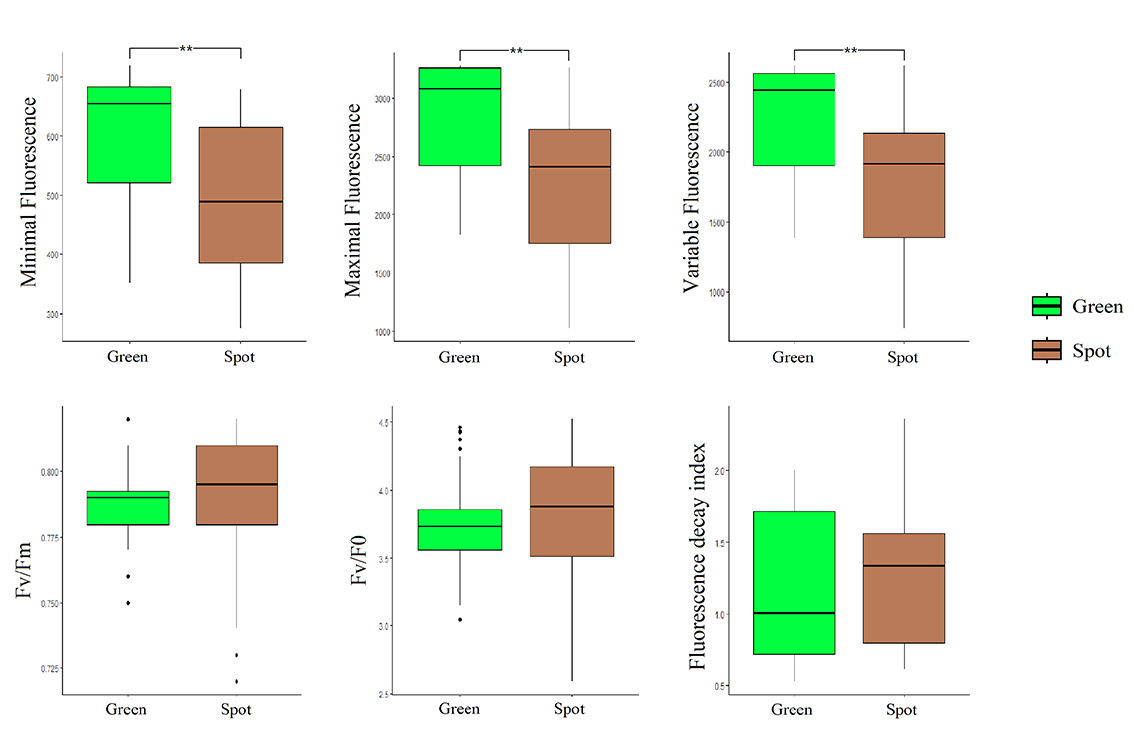

Supplement: Supplementary file 4 [file Image_4.JPEG]

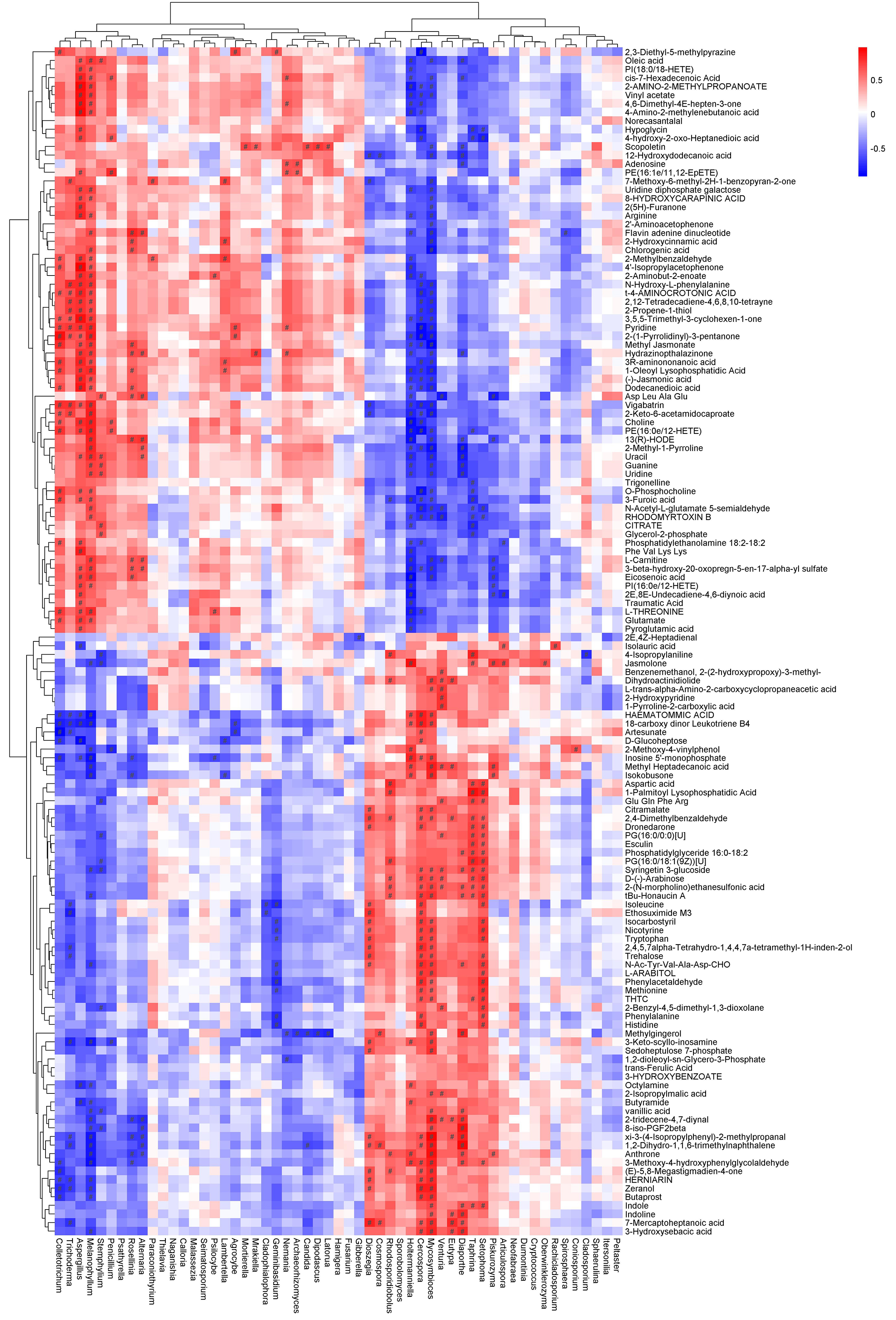

Supplement: Supplementary file 5 [file Image_5.JPEG]
