# Supplementary material for: Endosphere Microbiome and Metabolic Differences Between the Spots and Green Parts of Tricyrtis macropoda Leaves
Source: Front Microbiol. 2021 Jan 11;11:599829. doi: 10.3389/fmicb.2020.599829 (PMC7829350; doi:10.3389/fmicb.2020.599829)
Supplement: Supplementary file 6 [file Table_1.DOCX]

**Table S1**. Metabolites classification of *T. macropoda*

| **name** | **mode** | **trend** | **Class** |
| --- | --- | --- | --- |
| (-)-Jasmonic acid | － | ↓ | Organic acid |
| 12-Hydroxydodecanoic acid | － | ↓ | Organic acid |
| 13(R)-HODE | － | ↓ | Other |
| 1-Oleoyl Lysophosphatidic Acid | － | ↓ | Organic acid |
| 2-(1-Pyrrolidinyl)-3-pentanone | + | ↓ | Ketone |
| 2(5H)-Furanone | + | ↓ | Ketone |
| 2,12-Tetradecadiene-4,6,8,10-tetrayne | + | ↓ | Other |
| 2,3-Diethyl-5-methylpyrazine | + | ↓ | Terpene |
| 2-AMINO-2-METHYLPROPANOATE | + | ↓ | Lipid |
| 2'-Aminoacetophenone | + | ↓ | Ketone |
| 2-Aminobut-2-enoate | + | ↓ | Lipid |
| 2E,8E-Undecadiene-4,6-diynoic acid | + | ↓ | Organic acid |
| 2-Hydroxycinnamic acid | + | ↓ | Organic acid |
| 2-Keto-6-acetamidocaproate | + | ↓ | Other |
| 2-Methyl-1-Pyrroline | + | ↓ | Heterocycle |
| 2-Methylbenzaldehyde | + | ↓ | Aldehydes |
| 2-Propene-1-thiol | + | ↓ | Alcohols |
| 3,5,5-Trimethyl-3-cyclohexen-1-one | + | ↓ | Ketone |
| 3-beta-hydroxy-20-oxopregn-5-en-17-alpha-yl sulfate | － | ↓ | Other |
| 3-Furoic acid | + | ↓ | Organic acid |
| 3R-aminononanoic acid | + | ↓ | Organic acid |
| 4,6-Dimethyl-4E-hepten-3-one | + | ↓ | Ketone |
| 4-Amino-2-methylenebutanoic acid | + | ↓ | Organic acid |
| 4-hydroxy-2-oxo-Heptanedioic acid | + | ↓ | Organic acid |
| 4'-Isopropylacetophenone | + | ↓ | Ketone |
| 7-Methoxy-6-methyl-2H-1-benzopyran-2-one | + | ↓ | Ketone |
| 8-HYDROXYCARAPINIC ACID | － | ↓ | Organic acid |
| Adenosine | － | ↓ | Glycoside |
| Arginine | + | ↓ | Amino acid |
| Asp Leu Ala Glu | － | ↓ | Other |
| Chlorogenic acid | － | ↓ | Organic acid |
| Choline | + | ↓ | Alkaloid |
| cis-7-Hexadecenoic Acid | － | ↓ | Organic acid |
| CITRATE | － | ↓ | Salt |
| Dodecanedioic acid | － | ↓ | Organic acid |
| Eicosenoic acid | － | ↓ | Organic acid |
| Flavin adenine dinucleotide | － | ↓ | Organic acid |
| Glutamate | + | ↓ | Salt |
| Glycerol-2-phosphate | － | ↓ | Organic acid |
| Guanine | + | ↓ | Purine |
| Hydrazinopthalazinone | + | ↓ | Ketone |
| Hypoglycin | + | ↓ | Amino acid |

**Table S1.** Continued

| **name** | **mode** | **trend** | **Class** |
| --- | --- | --- | --- |
| L-Carnitine | + | ↓ | Alkaloid |
| L-THREONINE | + | ↓ | Amino acid |
| Methyl Jasmonate | － | ↓ | Lipid |
| N-Acetyl-L-glutamate 5-semialdehyde | + | ↓ | Aldehydes |
| N-Hydroxy-L-phenylalanine | + | ↓ | Amino acid |
| Norecasantalal | + | ↓ | Other |
| Oleic acid | － | ↓ | Organic acid |
| O-Phosphocholine | + | ↓ | Alkaloid |
| PE(16:0e/12-HETE) | － | ↓ | Other |
| PE(16:1e/11,12-EpETE) | － | ↓ | Other |
| Phosphatidylethanolamine 18:2-18:2 | － | ↓ | Other |
| PI(16:0e/12-HETE) | － | ↓ | Other |
| PI(18:0/18-HETE) | － | ↓ | Other |
| Pyridine | + | ↓ | Heterocycle |
| Pyroglutamic acid | + | ↓ | Amino acid |
| RHODOMYRTOXIN B | － | ↓ | Alkaloid |
| Scopoletin | + | ↓ | Other |
| t-4-AMINOCROTONIC ACID | + | ↓ | Organic acid |
| Traumatic Acid | － | ↓ | Organic acid |
| Trigonelline | + | ↓ | Alkaloid |
| Uracil | + | ↓ | Heterocycle |
| Uridine | － | ↓ | Glycoside |
| Uridine diphosphate galactose | － | ↓ | Carbohydrate |
| Vigabatrin | + | ↓ | Organic acid |
| Vinyl acetate | + | ↓ | Lipid |
| (E)-5,8-Megastigmadien-4-one | + | ↑ | Ketone |
| 1,2-Dihydro-1,1,6-trimethylnaphthalene | + | ↑ | Alkanes |
| 1,2-dioleoyl-sn-Glycero-3-Phosphate | － | ↑ | Organic acid |
| 18-carboxy dinor Leukotriene B4 | － | ↑ | Olefin |
| 1-Palmitoyl Lysophosphatidic Acid | － | ↑ | Organic acid |
| 1-Pyrroline-2-carboxylic acid | + | ↑ | Organic acid |
| 2-(N-morpholino)ethanesulfonic acid | + | ↑ | Organic acid |
| 2,4,5,7alpha-Tetrahydro-1,4,4,7a-tetramethyl-1H-inden-2-ol | + | ↑ | Other |
| 2,4-Dimethylbenzaldehyde | + | ↑ | Aldehydes |
| 2-Benzyl-4,5-dimethyl-1,3-dioxolane | + | ↑ | Heterocycle |
| 2E,4Z-Heptadienal | + | ↑ | Olefin |
| 2-Hydroxypyridine | + | ↑ | Heterocycle |
| 2-Isopropylmalic acid | － | ↑ | Organic acid |
| 2-Methoxy-4-vinylphenol | + | ↑ | Phenols |
| 2-tridecene-4,7-diynal | + | ↑ | Aldehydes |
| 3-HYDROXYBENZOATE | － | ↑ | Lipid |
| 3-Hydroxysebacic acid | － | ↑ | Organic acid |

**Table S1.** Continued

| **name** | **mode** | **trend** | **Class** |
| --- | --- | --- | --- |
| 3-Keto-scyllo-inosamine | + | ↑ | Polysaccharide |
| 3-Methoxy-4-hydroxyphenylglycolaldehyde | + | ↑ | Aldehydes |
| 4-Isopropylaniline | + | ↑ | Amine |
| 7-Mercaptoheptanoic acid | + | ↑ | Organic acid |
| 8-iso-PGF2beta | － | ↑ | Other |
| Anthrone | + | ↑ | Ketone |
| Artesunate | － | ↑ | Lipid |
| Aspartic acid | + | ↑ | Amino acid |
| Benzenemethanol, 2-(2-hydroxypropoxy)-3-methyl | + | ↑ | Alcohols |
| Butaprost | － | ↑ | Other |
| Butyramide | + | ↑ | Amine |
| Citramalate | － | ↑ | Organic acid |
| D-(-)-Arabinose | － | ↑ | Carbohydrate |
| D-Glucoheptose | － | ↑ | Carbohydrate |
| Dihydroactinidiolide | + | ↑ | Heterocycle |
| Dronedarone | － | ↑ | Other |
| Esculin | － | ↑ | Glycoside |
| Ethosuximide M3 | + | ↑ | Oxime |
| Glu Gln Phe Arg | － | ↑ | Other |
| HAEMATOMMIC ACID | － | ↑ | Organic acid |
| HERNIARIN | + | ↑ | Other |
| Histidine | + | ↑ | Amino acid |
| Indole | + | ↑ | Heterocycle |
| Indoline | + | ↑ | Heterocycle |
| Inosine 5'-monophosphate | － | ↑ | Organic acid |
| Isocarbostyril | + | ↑ | Lipid |
| Isokobusone | － | ↑ | Other |
| Isolauric acid | － | ↑ | Organic acid |
| Isoleucine | + | ↑ | Amino acid |
| Jasmolone | + | ↑ | Ketone |
| L-ARABITOL | － | ↑ | Polysaccharide |
| L-trans-alpha-Amino-2-carboxycyclopropaneacetic acid | + | ↑ | Organic acid |
| Methionine | + | ↑ | Amino acid |
| Methyl Heptadecanoic acid | － | ↑ | Organic acid |
| Methylgingerol | － | ↑ | Phenols |
| N-Ac-Tyr-Val-Ala-Asp-CHO | － | ↑ | Other |
| Nicotyrine | + | ↑ | Alkaloid |
| Octylamine | + | ↑ | Amine |
| PG(16:0/0:0)[U] | － | ↑ | Other |
| PG(16:0/18:1(9Z))[U] | － | ↑ | Other |
| Phenylacetaldehyde | + | ↑ | Aldehydes |
| Phenylalanine | － | ↑ | Organic acid |

**Table S1.** Continued

| **name** | **mode** | **trend** | **Class** |
| --- | --- | --- | --- |
| Phosphatidylglyceride 16:0-18:2 | － | ↑ | Lipid |
| Sedoheptulose 7-phosphate | － | ↑ | Carbohydrate |
| Syringetin 3-glucoside | － | ↑ | Glucoside |
| tBu-Honaucin A | + | ↑ | Other |
| THTC | + | ↑ | Other |
| trans-Ferulic Acid | + | ↑ | Organic acid |
| Trehalose | － | ↑ | Carbohydrate |
| Tryptophan | － | ↑ | Amino acid |
| vanillic acid | － | ↑ | Organic acid |
| xi-3-(4-Isopropylphenyl)-2-methylpropanal | + | ↑ | Alkanes |
| Zeranol | － | ↑ | Alkaloid |
