# Supplementary material for: Endosphere Microbiome and Metabolic Differences Between the Spots and Green Parts of Tricyrtis macropoda Leaves
Source: Front Microbiol. 2021 Jan 11;11:599829. doi: 10.3389/fmicb.2020.599829 (PMC7829350; doi:10.3389/fmicb.2020.599829)
Supplement: Supplementary file 7 [file Table_2.DOCX]

**Table S2:** The endophytic fungi of classification significantly related

| **Phylum** | **Class** | **Order** | **Family** | **Genus** |
| --- | --- | --- | --- | --- |
| Basidiomycota | Tremellomycetes | Holtermanniales | Holtermanniales | Holtermanniella |
| Ascomycota | Dothideomycetes | Capnodiales | Mycosphaerellaceae | Cercospora |
| Ascomycota | Leotiomycetes | Helotiales | Helotiaceae | Mycosymbioces |
| Ascomycota | Sordariomycetes | Diaporthales | Diaporthaceae | Diaporthe |
| Basidiomycota | Tremellomycetes | Tremellales | Bulleribasidiaceae | Dioszegia |
| Ascomycota | Sordariomycetes | Hypocreales | Nectriaceae | Cosmospora |
| Ascomycota | Dothideomycetes | Venturiales | Venturiaceae | Venturia |
| Ascomycota | Sordariomycetes | Xylariales | Diatrypaceae | Eutypa |
| Basidiomycota | Microbotryomycetes | Sporidiobolales | Sporidiobolaceae | Rhodosporidiobolus |
| Ascomycota | Taphrinomycetes | Taphrinales | Taphrinaceae | Taphrina |
| Ascomycota | Dothideomycetes | Pleosporales | Phaeosphaeriaceae | Setophoma |
| Basidiomycota | Tremellomycetes | Filobasidiales | Piskurozymaceae | Piskurozyma |
| Basidiomycota | Microbotryomycetes | Microbotryomycetes | Chrysozymaceae | Oberwinklerozyma |
| Ascomycota | Leotiomycetes | Helotiales | Helotiaceae | Articulospora |
| Ascomycota | Dothideomycetes | Capnodiales | Cladosporiaceae | Rachicladosporium |
| Ascomycota | Leotiomycetes | Helotiales | Helotiales | Spirosphaera |
| Ascomycota | Eurotiomycetes | Chaetothyriales | Herpotrichiellaceae | Coniosporium |
| Ascomycota | Sordariomycetes | Xylariales | Xylariaceae | Rosellinia |
| Ascomycota | Dothideomycetes | Pleosporales | Pleosporaceae | Alternaria |
| Ascomycota | Dothideomycetes | Pleosporales | Pleosporaceae | Stemphylium |
| Ascomycota | Eurotiomycetes | Eurotiales | Aspergillaceae | Penicillium |
| Ascomycota | Sordariomycetes | Glomerellales | Glomerellaceae | Colletotrichum |
| Ascomycota | Sordariomycetes | Hypocreales | Hypocreaceae | Trichoderma |
| Ascomycota | Eurotiomycetes | Eurotiales | Aspergillaceae | Aspergillus |
| Basidiomycota | Agaricomycetes | Agaricales | Agaricaceae | Melanophyllum |
| Ascomycota | Dothideomycetes | Pleosporales | Didymosphaeriaceae | Paraconiothyrium |
| Ascomycota | Leotiomycetes | Helotiales | Rutstroemiaceae | Lambertella |
| Basidiomycota | Agaricomycetes | Agaricales | Strophariaceae | Psilocybe |
| Basidiomycota | Agaricomycetes | Agaricales | Bolbitiaceae | Agrocybe |
| Mortierellomycota | Mortierellomycetes | Mortierellales | Mortierellaceae | Mortierella |
| Basidiomycota | Tremellomycetes | Cystofilobasidiales | Cystofilobasidiales | Mrakiella |
| Ascomycota | Sordariomycetes | Xylariales | Xylariaceae | Nemania |
| Ascomycota | Archaeorhizomycetes | Archaeorhizomycetales | Archaeorhizomycetaceae | Archaeorhizomyces |
| Ascomycota | Saccharomycetes | Saccharomycetales | Saccharomycetales | Candida |
| Ascomycota | Saccharomycetes | Saccharomycetales | Dipodascaceae | Dipodascus |
| Ascomycota | Dothideomycetes | Pleosporales | Pleosporales | Latorua |
| Ascomycota | Eurotiomycetes | Chaetothyriales | Herpotrichiellaceae | Cladophialophora |
| Basidiomycota | Geminibasidiomycetes | Geminibasidiales | Geminibasidiaceae | Geminibasidium |
| Ascomycota | Dothideomycetes | Capnodiales | Cladosporiaceae | Cladosporium |
| Ascomycota | Sordariomycetes | Hypocreales | Nectriaceae | Gibberella |
